# Generation of a Biotin-Tagged Dual-Display Phage

**DOI:** 10.3390/cells13201696

**Published:** 2024-10-14

**Authors:** Laura Maria De Plano, Salvatore Oddo, David Bikard, Antonella Caccamo, Sabrina Conoci

**Affiliations:** 1 Department of Chemical, Biological, Pharmaceutical and Environmental Sciences, University of Messina, Viale F. Stagno d’Alcontres 31, 98166 Messina, Italy; lauramaria.deplano@unime.it (L.M.D.P.); salvatore.oddo@unime.it (S.O.); sabrina.conoci@unime.it (S.C.); 2 Pasteur Institute, University of Paris, Synthetic Biology, 75015 Paris, France; david.bikard@pasteur.fr; 3 Department of Chemistry G. Ciamician, University of Bologna, Via F. Selmi 2, 40126 Bologna, Italy; 4 LAB Sense Beyond Nano—DSFTM CNR, Viale F. Stagno d’Alcontres 31, 98166 Messina, Italy

**Keywords:** bacteriophage, phage display, bacteria, cloning, plasmid

## Abstract

Phage display is widely used in biomedical research. One of the great advantages of phage display is the specificity of the connection of a foreign peptide exposed outside the capsid to the intended target. Secondary detection systems, which are often laborious and costly, are required to identify and quantify the peptide/target interaction. In this study, we generated a novel dual-display phage to facilitate the detection and quantification of the peptide/target interaction. First, we generated a biotin-tagged phage by adding a small biotin-accepting peptide (sBT) to gene-3 of the M13K07 helper phage. Subsequently, we enhanced the M13K07 biotin-tagged phage by incorporating a selective peptide on gene-8, which is then exposed to the phage capsid. The exposed peptide acts as a probe to bind to a selective molecular target, whose interaction can be readily visualized thanks to the biotinylated phage. Our versatile dual-display phage exhibits high flexibility; by swapping the displayed peptide/probe, one can change the phage target while retaining the sBT gene in-frame with the pIII. We expect the generated biotin-tagged dual phages to be used as a multifunctional probe to couple with several streptavidin-biotin-based systems.

## 1. Introduction

The streptavidin-biotin system is routinely used for the detection, localization, purification and immobilization of nucleic acids, proteins and other macromolecules [1,2,3]. Its widespread application is mainly due to the high affinity between biotin and streptavidin. Though several biotinylation reagents are available, chemical biotinylation can result in the binding site’s inactivation or the incorrect labeling of the target protein [4,5]. To mitigate these issues, alternative approaches to covalently bind biotin to proteins of interest have been developed; for example, a target protein can be linked to a biotin acceptor peptide (BAP), which, in turn, is covalently linked to biotin [6]. BAPs of 13–15 amino acids are defined as small biotin targets (sBTs) and are regularly used because they are efficiently recognized by biotin holoenzyme synthetase (BHS), encoded by the BirA gene [7,8]. In this context, BAPs can be easily biotinylated both in vitro, using purified recombinant *E. coli* BirA enzyme, and in vivo by transfecting a plasmid expressing *E. coli* BirA in a selected phage [9]. The insertion of BAPs into a protein of interest allows the generation of a platform that can be used with several streptavidin-biotin-based systems. 

Phage display utilizes bacteriophages (or phages) to select foreign peptides exposed outside the phage capsid [10,11]. This method has been extensively applied in different aspects of biology [12,13,14,15,16,17,18]. M13, the phage mainly used in phage display, consists of a filamentous structure of 1 µm in length and 7 nm in diameter with a 6400 bp DNA genome [19]. The long cylindrical capsid is composed of approximately 2700 copies of the pVIII protein, and between three and five copies of minor coat proteins (pIII, pV, pVII and pIX). The immature pVIII comprises 73 amino acids; the first 23 constitute a signal peptide, which is eventually deleted leading to the mature pVIII. The immature pIII is made of 424 amino acids [20]. The N-terminal 18 amino acids code for a signal peptide lost in the mature pIII protein. The 406 amino acid residues are divided into two N-terminal domains (N1 and N2), linked by a glycine-rich region, which is critical for the phage infection, and a C-terminal domain responsible for the interaction with pVI and anchoring to the phage capsid [21].

Both pIII and pVIII have been manipulated so that the mature phage would expose external peptides [22]. Indeed, by genetically modifying the p3 and p8 genes, one can add a DNA coding sequence for a random peptide. By doing so, the random foreign peptide fused in-frame to the N-terminus of the pIII or pVIII capsid proteins is exposed on the outside of the phage capsid. Phage display uses two types of vectors, phage and phagemid vectors [23]. The phage vector is based on the M13 phage genome, in which either gene-3 or gene-8 is fused to foreign DNA sequences encoding the peptide of interest [24]. The phagemid vector is a plasmid expressing only one capsid protein modified with the foreign sequence and carrying a packaging signal. Consequently, the phagemid vector needs a helper phage (i.e., phage vector such as M13 or M13-derived), which carries all the other capsid proteins needed to generate a functional phage progeny [23].

In this work, we generated a biotin-tagged dual display phage (herein referred to as DDP) by transfecting *E. coli* with three different plasmids: (i) a plasmid harboring the BirA gene; (ii) the phagemid vector expressing a peptide fused to gene-8; and (iii) a phage vector harboring the modified gene-3 with the sBT sequence. By doing so, we obtained a phage exposing a peptide of interest on pVIII and a biotin on pIII. This system will allow us to easily identify the interaction between phage/target through a simple avidin–biotin interaction.

## 2. Materials and Methods

### 2.1. Bacterial Strains

For cloning and plasmid construction, we used *E. coli* MG1655. For phage amplification, we used *E. coli* TG1. Both *E. coli* strains were propagated in the Luria Bertani broth (LB) and Agar (LA) substrates and kept at −80 °C in 20% glycerol.

### 2.2. Bacteriophage and Plasmid

The M13K07 phage was employed as the structural basis for the cloning reactions. The M13K07 helper phage harbors a gene that confers resistance to kanamycin (Kan+) and a p15A origin of replication. The M13K07 phage is routinely employed as a helper phage in coinfecting *E. coli* with a phagemid vector to generate fully functional phages.

Li5 phagemid vectors, which display the foreign peptide RKILRAGPL in-frame on the pVIII protein, were employed to create the DDP [25,26]. The Li5 phagemid vectors contain an origin of replication, the DNA packaging region from the M13 phage, a gene that confers resistance to ampicillin, and the nucleotide sequence coding for the foreign peptide in-frame to gene-8. Additionally, upstream and downstream of gene-8, there are the isopropyl ß-D-1-thiogalactopyranoside (IPTG) promoter and the LacZ gene, respectively. Together with a helper phage (e.g., M13K07), these vectors are used to generate a new phage with a phenotype resembling an M13 phage. Phages were maintained in TBS (7.88 g/L of Tris HCl, and 8.77 g/L of NaCl in dH_2_O). p-CM-tetR, containing chloramphenicol (CM+) and the tet-R promoter, was kept in *E. coli* MG1655.

### 2.3. Construction of New Genome MK-sBT-3

The cloning steps to generate the MK-sBT-3 vector were performed as previously described [27]. Briefly, the DNA templates were obtained from M13K07, or p-CM-tetR plasmids, which were isolated using the QIAprep Spin Miniprep Kit (Qiagen, Hilden, Germany). The primers used to perform a Phusion PCR are reported in Table 1. Approximately 10 ng of DNA template were used in a PCR mixture containing 10 µL of 5X HF buffer, 1 μL of dNTPs mix (10 mM each), 2.5 μL of each primer and 0.5 μL of Phusion^®^ High-Fidelity DNA polymerase. Quantities of 5 μL of amplified fragments were analyzed by gel electrophoresis (1% *w*/*v* agarose in 1X TAE buffer), purified by Nucleospin Gel and PCR clean-up (Qiagen) and quantified using Nanodrop. The isolated fragments were combined using the Gibson assembly method [28]. Tubes were incubated at 50 °C for 1 h. Subsequently, the product of the reaction was purified and used to transform 20 µL of competent *E. coli*. The transformed cells were then incubated for 1 h at 37 °C with 1 mL SOC on an orbital shaker at 200 rpm. After this incubation period, cells were plated on LA with Kan, or chloramphenicol based on the performed cloning. For each cloning, to check the correct newly added sequences, thirteen colonies were selected and grown in LB media with appropriate antibiotics at 37 °C. Subsequently, we added 50 µL ddH_2_O to the media before performing colony PCR (primers used are listed in Table 1). PCR amplicons were purified by NucleoSpin PCR Clean-up (Macherey–Nagel) and sequenced.

### 2.4. Formation of Viable MK-sBT-3 Phage

The transformed colonies with MK-sBT-3 were selected and inoculated in LB with the appropriate antibiotic. Cultures were grown through the night at 37 °C in an orbital shaker. To collect the phages, we followed a well-established approach [29]. Briefly, we centrifuged the culture at 8000× *g* for 20 min, removed the supernatant and mixed it with 25% PEG/NaCl. The mixture was then incubated on ice for 4 h, after which it was centrifuged at 15,300× *g* for an additional hour at 4 °C. We then resuspended the pellet in 10% TBS and 25% (*v*/*v*) of PEG/NaCl. After being incubated for 4 h on ice, the mixture was centrifuged as described above. We then resuspended the pellet, which contained the phage particles, in 10% TBS before filtering it using a membrane with a pore size of 0.22 μm. The generated phage particles were kept at 4 °C until use. A quantity of 10 μL of the phage-containing solution was used to infect *E. coli* to assess bacterial growth. Specifically, first *E. coli* was incubated with the phages at 37 °C for 15 min followed by 20 min incubation under gentle agitation. Subsequently, samples were dispensed into LA plates containing the proper antibiotic and incubated at 37 °C overnight.

### 2.5. M13K07 and MK-sBT-3 Phage Vector Propagation

The *E. coli* strain TG1 infected with the MK-sBT-3 vector or the wild-type M13K07 vector was inoculated into 20 mL LB containing 50 μg/mL kanamycin. We then incubated the mixture at 37 °C while shaking at 250 rpm. When the OD600 reading of the LB reached 0.2, the solution was transferred to 250 mL LB containing 50 μg/mL kanamycin and incubated at 37 °C overnight. 

For in vivo biotinylation, *E.coli* TG1 harboring p-BirA was used to perform the infection with MK-sBT-3 or wild-type M13K07 vector. Subsequently, cells were grown in kanamycin and chloramphenicol. Moreover, tetracycline (25 µg/mL) and d-biotin were added to the broth culture before overnight incubation.

### 2.6. Generation of the DDP

*E. coli* TG1 was exposed to the Li5 vector and cells were first grown for 15 min at 37 °C, without shaking, and then for an additional 20 min at 250 rpm. Following these incubation steps, *E. coli* was spread on LA plates, prepared with the proper antibiotic, and left overnight at 37 °C. We then selected a colony containing the phagemid vector (resistant to ampicillin) and inoculated it into 20 mL of LB supplied with the proper antibiotic. When the OD600 reading of the LB reached 0.2, 40 μg/mL IPTG and the MK-sBT-3 helper phage were added. We then incubated everything for 20 min at 37 °C. The culture was subsequently incubated at 37 °C overnight with 250 mL LB enriched with kanamycin and ampicillin. Additionally, we quantified the phages by titering (TU/mL) following a procedure reported in [29]. For in vivo biotinylation, we followed the same procedure described below.

### 2.7. In Vitro Biotinylation

A quantity of 236 μL of phage was biotinylated by the Enzymatic Protein Biotinylation Kit (Catalog Number CS0008) following the manufacturer’s instructions. The phage was quantified by Nanodrop and converted in mg/mL, assuming that each 0.38 absorbance was equivalent to 0.1 mg mL^−1^.

### 2.8. Biotin Binding Assay

Quantities of 100 μL of suspensions of biotinylated MK-sBT-3 and M13K07 (1012 TU/mL) were added to 96-well plates and grown through the night at 4 °C. The plates were then washed with 0.05% Tween 20 in PBS and blocked with 6% non-fat dry milk, and 0.05% Tween 20 in PBS for 2 h at 37 °C. After blocking, plates were washed five times and incubated with 100 μL streptavidin-HRP diluted at 1:200 in 1% non-fat dry milk, and 0.05% Tween 20 in PBS. After 1 h incubation at 37 °C, plates were washed five times before developing with 100 μL TMB for 45 min at room temperature. To stop the reaction, we added 100 μL of 1M H_2_SO_4_. M13K07 wild-type phage particles were used as a negative control for the evaluation of background from nonspecific binding.

### 2.9. Phage Binding Assay

A quantity of 100 μL *E. coli* suspension in exponential growth was coated on 96-well plates through the night at 4 °C in 15 mM Na_2_CO_3_, 35 mM NaHCO_3_, at pH 9.6. The plates were then washed 3X in 0.05% Tween 20 in PBS. The nonspecific binding sites were blocked for 2 h at 37 °C by incubating the plates as described in Section 2.8. After blocking, plates were washed five times and incubated at 37 °C with 100 μL of phage (Li5 and biotinylated Li5-MK-sBT-3 or 1012 TU/mL) between 15 and 240 min while shaking. After the incubation process, plates were re-washed 5X again and re-incubated for 1 h at 37 °C with 100 μL of anti-pVIII-M13-HRP or streptavidin-HRP, which were diluted 1:2500 and 1:200, respectively. After incubation and five additional washes, we added 100 μL TMB to develop the reaction. After 45 min of incubation with TMB, we stopped the development reaction with 100 μL of 1 M H_2_SO_4_.

### 2.10. DDP Binding Assay in Co-Culture

We individually cultured *P. aeruginosa* and *E. coli* F- in LB media at 37 °C in agitation overnight. The cultures were diluted in carbonate buffer (35 mM NaHCO_3_, 15 mM Na_2_CO_3_, pH 9.6) and counted by Colony Forming Units (CFUs) in LA agar plates. Specifically, 100 μL *E. coli* F- at decreasing concentrations (1 × 10^5^; 1 × 10^4^; 1 × 10^3^; 1 × 10^2^ CFU/mL) and 100 μL *P. aeruginosa* at 10^5^ CFU/mL in carbonate buffer (35 mM NaHCO_3_, 15 mM Na_2_CO_3_, pH 9.6), were coated alone or in combination on 96-well plates and incubated overnight at 4 °C. The plates were then washed once with 0.05% Tween 20 in PBS, and the nonspecific binding sites were blocked with 6% non-fat dry milk and 0.05% Tween 20 in PBS for 2 h at 37 °C. After blocking, the plates were washed once and incubated at 37 °C with 100 μL of DDP (biotinylated Li5-MK-sBT-3 at 10^13^ TU/mL) for 1 h with shaking. After this incubation, the plates were washed five times and re-incubated for 1 h at 37 °C with 100 μL of streptavidin-ALP (alkaline phosphatase) at a dilution of 1:500. Following incubation and five additional washes, 130 μL of the substrate p-nitrophenyl phosphate (pNPP) was added to develop the reaction. After 1 h of incubation at room temperature in the dark, the reaction was stopped with 20 μL of stop solution.

## 3. Results

**Figure 1:**
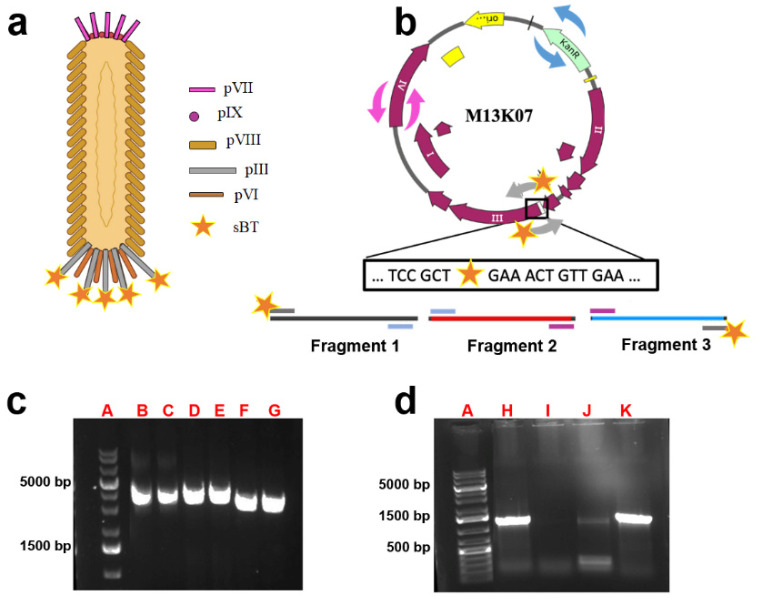
Generation of Mk-sBT-3 vectors. (**a**) Schematic representation of the M13 bacteriophage, emphasizing the capsid proteins. (**b**) Schematic structures of the ssDNA M13K07 helper phage. The orange star indicates the precise locations where the sBT sequence (LASIFEAQKIEWR) will be cloned. The arrows inside and outside the plasmid indicate the position of the 3 sets of primers used. The primers indicated with the gray arrows have a 5′ overhand (orange star) with the sBT sequence. The predictive fragments derived from the PCR are indicated as Fragments 1–3. (**c**) The panel reports the fragments obtained by PCR and separated by agarose electrophoresis (A: DNA marker, B and C: Fragment 1, D and E: Fragment 2, F and G: Fragment 3). (**d**) Electrophoresis of PCR colonies obtained to confirm the presence of the sBT sequence within the newly generated phages (A: DNA marker; H and K indicate the presence of the sBT sequence in the reconstituted M13K07 vector; I and J indicate colonies that did not incorporate the sBT sequence). The DNA from the amplicon in lane K was isolated and sequenced.

To generate a biotinylated phage, we used M13K07 as an initial step. We cloned the sequence coding for a thirteen amino acid sBT (LASIFEAQKIEWR 9) within gene-3 of M13K07, which codes for the capsid protein pIII (Figure 1a). The pVIII protein is central to maintaining the phage shell and integrity, while pIII is important for infectivity and stability of the particle as well as packaging and lysis of the host with consequent phage release [21,30].

To preserve the phage structure and ensure the integrity of the entire phage particle, we incorporated the sBT sequence downstream of the start codon of the mature protein (as indicated by the orange star in Figure 1b). Specifically, to clone the sBT sequence within gene-3 of M13K07, we designed three sets of primers, each with specific overhangs at the N-terminus of 18–27 base pairs; Table 1). Two of these primers carry the 39 base-pair-long sBT sequence tail at the 5′ end (Figure 1b). After PCR amplification, we obtained three fragments named Fragment-1, Fragment-2 and Fragment-3 of about 3 Kb each (Figure 1c and Table 2). The nucleotide sequence of each primer’s overhang aligns perfectly with the overhang of the primer employed to expand the neighboring fragment. For instance, Fragment-1 was amplified by employing a primer containing an sBT gene at its 5′-end and an overhang complementary to the 5′ overhang of the reverse primer employed for Fragment-3 (Figure 1b). Subsequently, we employed the Gibson assembly reaction to fuse together the PCR products (Fragments 1, 2 and 3; Table 2). We used the ligation products to transform *E. coli* and selected the recombinant clones by the appropriate antibiotic resistance. We confirmed the presence of the sBT sequence by PCR and found that two of the selected clones derived from the M13K07 backbone had a band corresponding to the presence of the sBT sequence (Figure 1d). We confirmed the sequencing of the helper phage vector with the sBT sequence (hereafter called MK-sBT-3) by sequencing.

**Figure 2:**
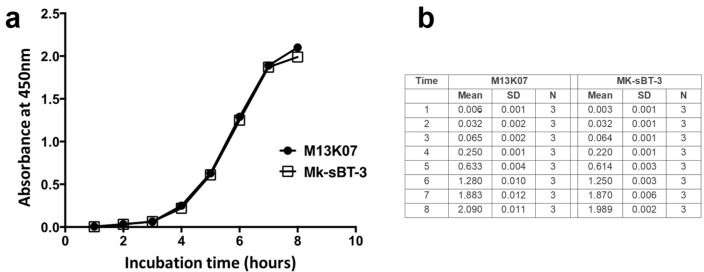
Growth rate of *E. coli* infected with Mk-sBT-3. (**a**) The graph displays *E. coli* growth after it was infected with Mk-sBT-3 or M13K07. The cultures were monitored every hour for 8 h by absorbance at 450 nm. (**b**) Mean and standard deviation of each time point for the two groups. Paired *t*-test indicates no significant difference for both samples with respective wild-type control.

To generate engineered phage particles from the MK-sBT-3 phage vector, we simply transformed *E. coli* with MK-sBT-3 phage. We then performed amplification and precipitation steps (see experimental procedures) to isolate the phage particles. Subsequently, we infected *E. coli* with the newly generated phage particles and assessed the bacterial growth. We found that the MK-sBT-3 phage did not interfere with *E. coli* growth as indicated by the similar growth rates between bacteria infected with MK-sBT-3 and wild-type M13K07 phages (Figure 2). To measure the amount of phage produced by the infected bacteria, we measured the titer of their supernatant and found that the number of viruses generated was similar between the wild-type and engineered phage particles (Table 3). Taken together, these data indicate that the presence of the sBT sequence with the MK-sBT-3 phages did not alter their capability to infect *E. coli* and generate functional phages.

**Figure 3:**
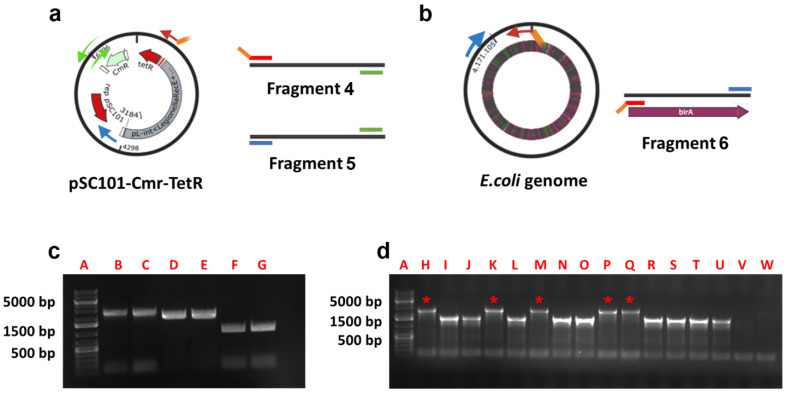
Generation of p-BirA plasmid. (**a**,**b**) Cartoon representation of the genome structures used to clone the BirA gene from the *E. coli* genome into the pSC101 plasmid. The predictive fragments obtained from the PCR are indicated as Fragments 4, 5 and 6. (**c**) Agarose electrophoresis gel of the PCR products indicates the bands generated (A: DNA marker, B and C: Fragment 6, D and E: Fragment 7, F and G: Fragment 8. (**d**) Electrophoresis gel of 16 PCR colonies was performed to verify the integration of BirA. Positive colonies are indicated with red asterisks. The DNA from the amplicon in lane K was isolated and sequenced.

We next sought to determine whether the sBT sequence within gene-3 was functional. To do so, we biotinylated the MK-sBT-3 phage vector in vitro, using a commercially available kit, and in vivo, in an *E. coli* host strain. To perform the biotinylation in vivo, we cloned the *E. coli* BirA gene under the control of a pTET promoter on an ori-pSC101 plasmid harboring a chloramphenicol resistance gene (CmR). Specifically, we designed three sets of primers, each with 5′ overhangs of 30–38 base pairs (Table 1). Two primer pairs were used to amplify the plasmid; we named the PCR products obtained by this amplification Fragment-4 and Fragment-5 (Figure 3a–c; Table 2). The last primer’s set was employed to expand the BirA gene from the *E. coli* genome; we named the product of this PCR Fragment-6 (Figure 3b,c). To ligate the PCR products, we employed the Gibson assembly and used the newly generated plasmids to transfect *E. coli*. The chloramphenicol-resistant clones were verified for the presence of the BirA gene by PCR colony screening. Of the clones analyzed, five contained a band matching the presence of the BirA gene (red asterisks in Figure 3d). We validated the correct structure of the new plasmid with the BirA gene (hereafter called p-BirA) by sequencing. 

**Figure 4:**
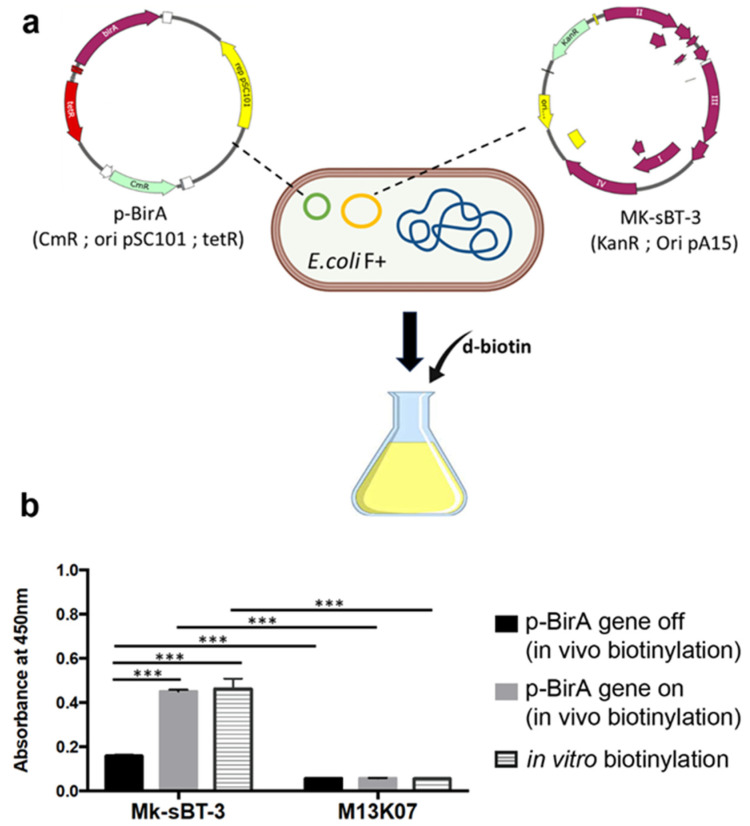
Validation of in vivo and in vitro phage biotinylation. (**a**) *E. coli* harboring plasmid-BirA was infected with the M13k07 helper phage. Bacteria were then grown in LB media supplemented with biotin. (**b**) Mk-sBT-3 phages were detected by a streptavidin-HRP assay after in vitro and in vivo biotinylation. The results indicate that both biotinylation systems are comparable. Consistent with the design of the whole experiment, keeping the BirA gene off does not produce any signal. *** indicates *p* < 0.0001.

We next determined whether *E. coli* carrying p-BirA could be used to biotinylate MK-sBT-3 in vivo. Specifically, we introduced the new p-BirA plasmid into *E. coli*, with the MK-sBT-3 phage, thereby obtaining new phages with the pIII containing the sBT sequence (Figure 4a). We then induced the expression of BirA by adding anhydrotetracycline to the bacterial culture during the phage amplification. To check whether biotin can bind to the sBT sequence exposed by pIII, we used a standard streptavidin-HRP assay. Our results indicate that the biotinylated MK-sBT-3 phage showed an absorption value four times higher than the control M13K07 phage (Figure 4b). These results indicate that pIII is biotinylated in our assay, with a greater signal observed upon induction of BirA. To complement these findings, we biotinylated our newly developed phages in vitro, using a commercially available kit. We found that even after biotinylation of the phage in vitro, the absorbance was similar to that obtained by in vivo biotinylation (Figure 4b). These data indicate that both approaches can be used interchangeably to biotinylate our phage, based on the experimental needs of the investigator. Nevertheless, the in vivo biotinylation process is less expensive and produces biotinylated phage particles in a single-step process. 

**Figure 5:**
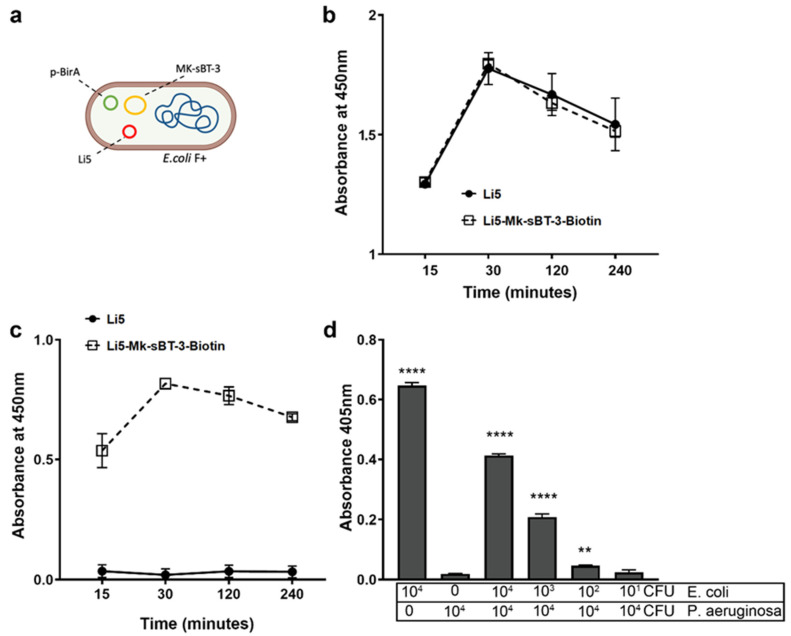
Examination of the biotinylated DDP system. (**a**) Schematic of the infection approach employed to make the biotinylated DDP. (**b**,**c**) Kinetics of the interaction phage/*E. coli* F- of the Li5 phage alone and the Li5-Mk-sBT-3 phage. In panel b, the interaction was detected by an anti-M13 antibody, while in panel c, it was detected by a streptavidin-HRP assay. T-test analysis showed a non-significant difference among the different points in panel a. In contrast, the signal between Li5 and Li5-Mk-sBT-3 was statistically significant at all time points in panel b (*p* < 0.0001). (**d**) Detection of *E. coli* by Li5-Mk-sBT-3 in a mixed culture of *E. coli* F- and *P. aeruginosa*. The interaction phage/target was detected by a streptavidin-ALP assay. The data reported are normalized to the system background signal. One-way ANOVA *p* < 0.0001. Tukey’s multiple comparisons: ** *p* = 0.0067; **** *p* < 0.0001 compared to *P. aeruginosa* alone.

We next tested the possibility of employing the MK-sBT-3 phage as a detection system when used with a phagemid construct engineered to identify a specific target. As a proof of concept, we combined the MK-sBT-3 phage with the Li5 phagemid whose pVIII had been engineered to display outside the capsid the foreign peptide RKILRAGPL [25]. We previously showed that the Li5 phage selectively interacts with *E. coli* [25]. We coinfected *E. coli* harboring pBirA with both Li5 and MK-sBT-3 phages, which led to the formation of a biotinylated DDP (Figure 5a). First, we assessed whether the biotinylated DDP retains the selectivity for the target *E. coli*. To do this, we monitored the kinetics of interaction between *E. coli* and the new phage particles. Our data indicate that the signal released was alike between the biotinylated DDP and the Li5 phage (Figure 5b). These data indicate that the peptide exposed on the pVIII has not been modified during the formation of the DDP system, which indeed maintains an identical binding effectiveness to the target. To test the biotinylated DDP, we measured the phage/target interaction with a streptavidin-HRP assay at various time points. After an incubation of 15 min, we could easily observe the biotinylated phage using the streptavidin-HRP signal, which was maintained through a 2 h incubation (Figure 5c). In contrast, the Li5 phage did not produce any signal as it did not bind streptavidin. These observations corroborate that the biotinylated DDP target bacteria because of the peptide fused to the pVIII, and the interaction is easily detectable with any streptavidin assay thanks to the sBT fused to the pIII, without the need for an application process or antibody. 

To further characterize our novel DDP, we investigated if it could recognize *E. coli* in a mixed bacterial culture. To this end, after we cultured *P. aeruginosa* and *E. coli* independently, we coated *P. aeruginosa* with decreasing concentrations of *E. coli* in 96-well plates and incubated the bacteria with the DDP. We used single cultures of *E. coli* and *P. aeruginosa* as positive and negative controls, respectively. As expected, the Li5-Mk-sBT-3 phage showed a robust signal in the *E. coli* single cultures and just a barely above-background signal for *P. aeruginosa* (Figure 5d). In the combined *E. coli/P. aeruginosa* wells, where the concentration of *P. aeruginosa* was kept constant at 1 × 10^4^ CFUs while the concentration of *E. coli* was gradually decreased, the signal decreased correspondingly with the concentration of *E. coli* (Figure 5d). One-way ANOVA with Tukey’s post hoc analyses indicated that we had to lower the concentration of *E. coli* by three orders of magnitude (to 10 CFUs) while keeping *P. aeruginosa* at 1 × 10^4^ CFUs before Li5-Mk-sBT-3 could not discriminate between the two bacterial strains. From these data, we calculated that the limit of detection (LOD), under these experimental conditions, was 100 CFUs of *E.coli*. Overall, these results indicated that Li5-Mk-sBT-3 also discriminated against the bacteria target in mixed cultures. 

## 4. Discussion

This study aimed to generate a DDP phage that would allow us to simultaneously detect a specific target through the engineered protein VIII and enable easy detection through the biotinylated protein III. Engineered phages have been employed in several fields and are often used as probes to detect specific molecular targets [17,31,32]. The identification and quantification of interactions between the foreign peptide exposed outside the phage capsid (the ligands or probe) and their targets (e.g., cells, proteins) are crucial steps. Traditionally, these interactions can be evaluated and quantified using several methods: (1) Enzyme-linked immunosorbent assay offers high sensitivity and specificity but may require antibodies against the target of interest, which might not always be readily available, and it may not be suitable for high-throughput screening. (2) Immunofluorescence assays allow for the visualization and localization of binding events at the cellular or subcellular level, but they might not be suitable for high-throughput screening or quantitative analysis. (3) Flow cytometry enables rapid and high-throughput analysis of binding interactions at the single-cell level. While it provides quantitative data and can be multiplexed for simultaneous analysis of multiple interactions, it requires specialized equipment and reagents. It may not be suitable for studying interactions occurring within subcellular compartments. (4) Surface plasmon resonance (SPR) allows for label-free, real-time monitoring of the phage/target interaction and provides quantitative data on binding kinetics and affinity. However, SPR instrumentation is expensive and requires expertise for operation and data analysis.

In this work, we generated a biotinylated DDP by (i) cloning an sBT sequence in-frame with gene-3, thereby creating the Mk-sBT-3 phage; and (ii) joining the Mk-sBT-3 phage with an external peptide attached to the N-terminus of pVIII. This peptide confers selective binding of the biotinylated DDP to target bacteria [25]. The sBT sequence on the pIII protein is conjugated with biotin and thus allows for easy detection and quantification of the phage/target interaction. Indeed, the streptavidin/biotin system represents a highly versatile approach that can be used in various experimental techniques, including protein purification, immunohistochemistry, western blotting, and enzyme-linked immunosorbent assays (ELISA) [4,33]. Its widespread use is primarily due to its high specificity and affinity. Streptavidin is a tetrameric protein obtained from *Streptomyces avidinii*, while biotin binds to streptavidin with a dissociation constant in the range of 10^−^^14^ to 10^−^^15^ M, making it one of the strongest non-covalent interactions known in nature. 

The ability to efficiently biotinylate phages in a site-specific region will open new prospects for their biotechnological application. Scholle and colleagues reported a system where a short random peptide library, expressed on pIII, is followed by an AviTag sequence [34]. Similarly, others have reported on the generation of phages in which pIII has been genetically modified to directly or indirectly bind biotin e.g., refs. [35,36]. In these cases, the biotinylated phages are captured by their interaction with streptavidin to perform affinity purification and downstream analyses. In contrast, our new system allows for the generation of multifunctional phage particles, whereby using targeted biotinylation one can selectively add biotin to one tip of the phage and expose a peptide/probe on the other phage tip. In addition to allowing us to perform affinity purification, as in the Scholle system, our newly developed phage can be used as a detection system as we showed here. Furthermore, this approach will allow for a more accurate development of in vitro detection systems. In other words, we can link the biotin-tagged DDP with a specific orientation to a solid support as the biotin is associated with the pIII. By doing so, the peptide/probe, which is associated with the pVIII, is unencumbered for the detection of a target. We expect that such a detection system will be more sensitive (as more free probes can interact with the target) and more reliable compared to a random passive absorption of phage to the detection surface.

Notably, our system is highly versatile and can be easily modified to identify a unique target of interest. Specifically, one could easily combine a phage of interest expressing a foreign peptide on pVIII with our MK-sBT-3 vector to obtain a biotinylated DDP able to recognize a specific target of interest. For example, we previously identified several phages that selectively target serum autoantibodies against amyloid-β, a peptide associated with the onset and progression of Alzheimer’s disease (AD) [26]. To detect and quantify the circulating autoantibodies after the phage/target interaction, we extracted the phage DNA and amplified it using real-time PCR [26]. By simply transfecting *E. coli* with p-BirA, Mk-sBT-3 and one of our AD phages (i.e., replacing the Li5 phage in Figure 5a with an AD phage), we can generate a DDP that recognizes AD autoantibodies with the engineered pVIII and can be easily detected with the biotinylated pIII.

## 5. Conclusions

Overall, this system can be modified by replacing the peptide on the pIII with another peptide specific to a desired target. We expect the generated biotin-tagged DDP to be used as a multifunctional probe to couple with several streptavidin-biotin-based systems. In addition, this new system can be easily integrated with several commercially available biotin/streptavidin assays, which do not require the use of specific antibodies as they are necessary when detecting phage/target interaction by ELISA. Overall, we anticipate that our biotinylated DDP system will be used for a variety of in vitro and in vivo applications expanding the use of multifunctional phages in the detection system where precise and regulated manipulation may be required.

## Figures and Tables

**Table 1:** List of the primers used.

Name		Sequence	Location
Fragment 1	LDP016 FW-3	CTAGCGTCTATCTTCGAGGCCCAAAAGATCGAGTGGCGAgaaactgttgaaagttgtttagcaaaac	M13K07 genome
	LDP018 RV-3	gaggatttagaagtattagactttacaaacaattcgacaac	
Fragment 2	LDP019 FW-4	agttgtcgaattgtttgtaaagtctaatacttctaaatcctc	
	LDP020 RV-4	tttatccgtactcctgatgatgcatggttactcacc	
Fragment 3	LDP021 FW-5	tggtgagtaaccatgcatcatcaggagtacggataaa	
	LDP017 RV-5	TCGCCACTCGATCTTTTGGGCCTCGAAGATAGACGCTAGagcggagtgagaatagaaaggaac	
Fragment 4	P1 FW-6	AAACGCCTGGTGCTACGCGGGTTCGAGAGCTCGCTTG	pSC101-Cmr-TetR
	LDP025 FW-5	tgcgtagtgcagaaaaataaCATGGTACGCGTGCTAGAGGCA	
Fragment 5	P2 FW-7	AAGCGAGCTCTCGAACCCGCGTAGCACCAGGCGTTTA	
	LDP026 FW-7	ggcacggtgttatccttcattcatTTTTGCCTCCTAACTAGGTCATTTGA	
Fragment 6	LDP027 FW-8	TAGTTAGGAGGCAAAAatgaatgaaggataacaccgtgccactgaaattgatt	*E.coli* genome DNA
	LDP028 FW-8	CCTCTAGCACGCGTACCATGttatttttctgcactacgcagggatatttcac	
	LDP022 RV	ccgccagcattgacaggag	Mk-sBT-3
	P3 FW	ctctgtagccgttgctacc	
	P4 FW	TGCGTAACGGCAAAAGCAC	p-BirA
	P5 FW	gtaacagatgaacagcatgtaacacc	

**Table 2:** Name, size and quantity of the fragments obtained during the cloning procedures.

Purified Amplicons Quantity			
	Size (bp)	ng/μL	Gibson Assembly
Fragment 1	3105	174.5	For phage vector Mk-sBT-3
Fragment 2	3039	197.5	
Fragment 3	2682	218.4	
Fragment 4	2508	180.85	For p-BirA
Fragment 5	2033	146.6	
Fragment 6	1006	72.54	

**Table 3:** Comparison of the phages generated.

Phage Name	Type	Display Foreign Peptide	Location	Titer (TU/mL)
M13K07	Phage	None	None	2.7 × 10^12^
Mk-sBT-3	Phage vector	sBT (Biotin acceptor)	pIII	2 × 10^12^

## Data Availability

The datasets used and/or analyzed during the current study are available from the corresponding author upon reasonable request.
